# Supplementary material for: The interactome of intact mitochondria by cross-linking mass spectrometry provides evidence for coexisting respiratory supercomplexes
Source: Mol Cell Proteomics. 2017 Dec 8;17(2):216–32. doi: 10.1074/mcp.RA117.000470 (PMC5795388; doi:10.1074/mcp.RA117.000470)
Supplement: Supplemental Data [file supp_RA117.000470_133922_0_supp_23978_fzffwf.zip › spectra_annotation/mito_DR_spectra_annotation/285-1-3-1-2-1.pdf]

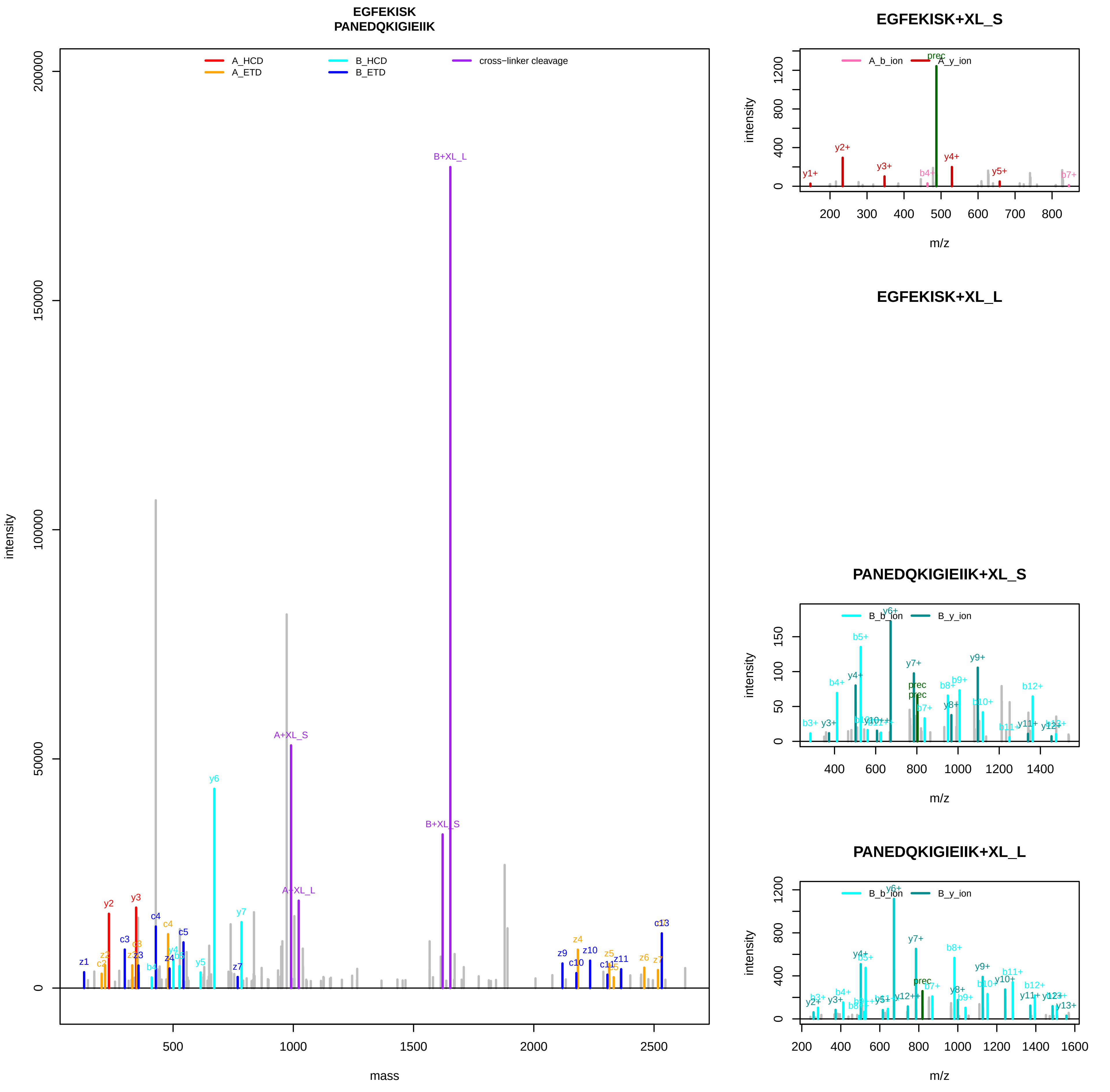
EGFEKISK
PANEDQKIGIEIIK
A_HCD
A_ETD
B_HCD
B_ETD
cross−linker cleavage
B+XL_L
A+XL_S
B+XL_S
A+XL_L
y2
y3
c3
c4
c5
y6
y7
y5
b4
z1
z7
z9
z10
c10
c11
z11
z4
z5
z6
c13
intensity
mass
0
50000
100000
150000
200000
500
1000
1500
2000
2500
EGFEKISK+XL_S
A_b_ion
A_y_ion
prec
y1+
y2+
y3+
b4+
y4+
y5+
b7+
m/z
EGFEKISK+XL_L
PANEDQKIGIEIIK+XL_S
B_b_ion
B_y_ion
y6+
b5+
y7+
y9+
b4+
y4+
b8+
b9+
b12+
y8+
b10+
b3+
y3+
y11+
y12+
PANEDQKIGIEIIK+XL_L
y6+
y7+
b8+
y9+
b11+
y10+
b12+
y13+
y2+
